# Growth differentiation factor-15 promotes glutamate release in medial prefrontal cortex of mice through upregulation of T-type calcium channels

**DOI:** 10.1038/srep28653

**Published:** 2016-06-29

**Authors:** Dong-Dong Liu, Jun-Mei Lu, Qian-Ru Zhao, Changlong Hu, Yan-Ai Mei

**Affiliations:** 1 Institutes of Brain Science, State Key Laboratory of Medical Neurobiology and School of Life Sciences, Fudan University, Shanghai 200433, China

## Abstract

Growth differentiation factor-15 (GDF-15) has been implicated in ischemic brain injury and synapse development, but its involvement in modulating neuronal excitability and synaptic transmission remain poorly understood. In this study, we investigated the effects of GDF-15 on non-evoked miniature excitatory post-synaptic currents (mEPSCs) and neurotransmitter release in the medial prefrontal cortex (mPFC) in mice. Incubation of mPFC slices with GDF-15 for 60 min significantly increased the frequency of mEPSCs without effect on their amplitude. GDF-15 also significantly elevated presynaptic glutamate release, as shown by HPLC. These effects were blocked by dual TGF-β type I receptor (TβRI) and TGF-β type II receptor (TβRII) antagonists, but not by a TβRI antagonist alone. Meanwhile, GDF-15 enhanced pERK level, and inhibition of MAPK/ERK activity attenuated the GDF-15-induced increases in mEPSC and glutamate release. Blocking T-type calcium channels reduced the GDF-15 induced up-regulation of synaptic transmission. Membrane-protein extraction and use of an intracellular protein-transport inhibitor showed that GDF-15 promoted Ca_V_3.1 and Ca_V_3.3 α-subunit expression by trafficking to the membrane. These results confirm previous findings in cerebellar granule neurons, in which GDF-15 induces its neurobiological effects via TβRII and activation of the ERK pathway, providing novel insights into the mechanism of GDF-15 function in cortical neurons.

Growth differentiation factor-15 (GDF-15), also known as GDF-15/MIC-1(macrophage inhibitory cytokine-1), is a member of the TGF-β superfamily. GDF-15 expression increases in response to tissue repair after acute injury, macrophage activation, cancer, and inflammation^1^,^2^,^3^,^4^. Increasing evidence suggests that GDF-15 is an integrative signal in pathology, with both adverse and beneficial effects, depending on the state of the cells and their microenvironment^5^. Better knowledge of the precise function and mechanism of action of GDF-15 is therefore needed to understand its activity and to further its development for clinical use.

Previous studies have indicated that GDF-15 is widely distributed in the central and peripheral nervous systems^6^. Strelau *et al*. (2009) showed that GDF-15-deficient mice exhibited progressive postnatal losses of spinal, facial, and trigeminal motor neurons, as well as sensory neurons in the dorsal root ganglia^7^. Recent researches found that recombinant GDF-15 enhanced hippocampal neural stem cell proliferation and neuronal differentiation^8^, repaired crushed optic nerve^9^, and aided axon regeneration following sciatic nerve crushing^10^, suggesting that GDF-15 might play survival-promoting and protective roles. Although GDF-15 is ubiquitous throughout the brain, its mRNA and protein levels are lower compared with those in the liver, lung, and kidney^11^. However, following injuries such as cryolesions or cerebral ischemic lesions in mice, GDF-15 mRNA and protein levels were dramatically up-regulated in neurons both at the lesion site and in neuronal populations projecting to the lesioned area^6^,^12^. However, although GDF-15 promotes survival and protects neurons against injury, the effects of high levels of GDF-15 on neuronal excitability and synaptic activity remain undetermined.

We recently demonstrated that GDF-15 increased the delayed rectifier outward K^+^ current and expression of its main component, K_V_2.1 α-subunit, by Src kinase activation via TβRII in rat granule neurons (CGNs)^13^. These data provided the first evidence that modulation of K^+^ channel expression and the downstream signaling pathways by GDF-15 was receptor- and non-Smad-dependent-pathway associated. However, whether the same signaling pathways and receptors identified in CGNs are activated by GDF-15 in other neurons remains unknown.

In this study, we evaluated the effects of GDF-15 on cortical neurons in slices of mouse medial prefrontal cortex (mPFC) and recorded miniature excitatory post-synaptic currents (mEPSCs) and T-type calcium channel currents (*I*_T-type VGCC_), while simultaneously measuring neurotransmitter glutamate release and membrane Ca^2+^ channel protein expression. We also determined if the same signaling pathways and TβRII receptor previously identified in CGNs were activated by GDF-15 in mPFC neurons under these conditions.

## Results

### GDF-15 increased mEPSC frequency in pyramidal neurons through activation of TGFβRII-mediated ERK1/2 pathway

**Figure 1:**
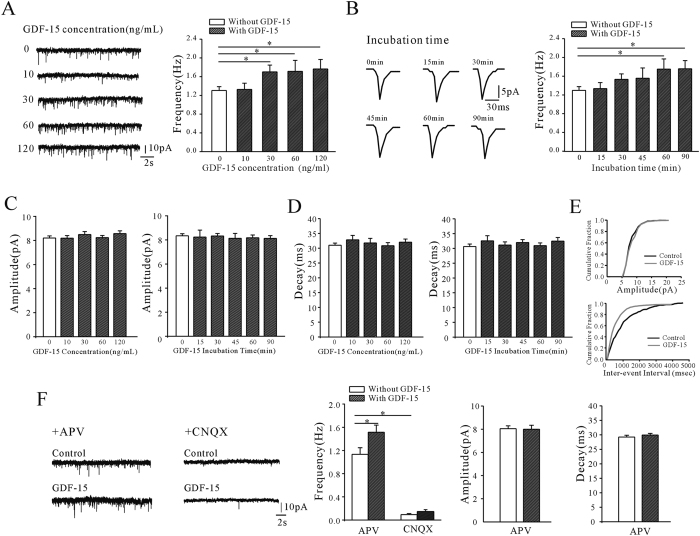
GDF-15 significantly increased the frequency but not amplitude of mEPSCs. (**A**) mEPSCs were recorded in control ACSF or ACSF with different concentrations of GDF-15 for 1 h. The left panel shows the representative recordings; the right panel shows the frequencies of mEPSCs under different concentrations of GDF-15. (**B**) mEPSCs were recorded in control ACSF or ACSF with 30 ng/mL of GDF-15 for different times. The left panel shows the representative averaged mEPSCs; the right panel shows the frequencies of mEPSCs under different incubation times of GDF-15, respectively. (**C**) mEPSC amplitude was unaffected by GDF-15, irrespective of concentration and incubation time. (**D**) mEPSC decay time was unaffected by GDF-15, irrespective of concentration and incubation time. (**E**) The upper panel shows the cumulative distributions of amplitudes and the bottom panel shows the cumulative distributions inter-event intervals. (**F**) the AMPA or NMDA receptors mediated mEPSC were separated by specific blockers. The left two panels show the representative recordings in presence of AMPA or NMDA receptors blockers; the right three panels show the frequencies, amplitudes and decay times of mEPSC in presence of AMPA or NMDA receptors blockers with or without GDF-15. Results are shown as means ± SEM. *p < 0.05 compared with corresponding control (without GDF-15) determined by one-way ANOVA.

We recorded mEPSCs from visually identified pyramidal neurons in mPFC layers II/III from cortex slices using whole-cell voltage clamping. mEPSCs were recorded at a holding potential of −70 mV in the presence of 1 μM TTX and 10 μM bicuculline to inhibit spontaneous action-potential generation and GABA_A_-mediated inhibitory postsynaptic currents, respectively. Incubation of mPFC slices with GDF-15 significantly increased the mEPSC frequency (Fig. 1A). Incubation of slices with 10, 30, 60, or 120 ng/ml GDF-15 for 1 h increased the mEPSC frequencies by 1.5% (from 1.30 ± 0.08 Hz of control to 1.32 ± 0.13 Hz, n = 39 and 19, *p* = 0.912), 30.8% (to 1.70 ± 1.45 Hz, n = 23, *p* = 0.034), 31.5% (to 1.71 Hz ± 0.24, n = 17, *p* = 0.048), and 35.4% (to 1.76 ± 0.20 Hz, n = 19, *p* = 0.022), respectively. The significant effect of GDF-15 (30 ng/ml) on mEPSC frequency occurred within the first 60 min. After incubation with GDF-15 (30 ng/mL) for 60 and 90 min, mEPSC frequencies increased by 34.8% and 35.7% (from 1.29 ± 0.08 Hz to 1.74 ± 0.22 Hz and 1.75 ± 0.17 Hz, n = 17 and 14, *p* = 0.012 and 0.017), respectively (Fig. 1B). However, increasing the concentration of GDF-15 from 10 to 120 ng/ml and prolonging the incubation time from 15 to 90 min did not affect the amplitude or decay time of the mEPSCs (Fig. 1C,D). The representative cumulative distributions of amplitude and inter-event interval are shown in Fig. 1E.

To investigate whether AMPA or NMDA receptors were involved in mEPSC in our study, APV (100 μM) or CNQX (25 μM) was used to block the NMDA or AMPA receptors, respectively. The mEPSCs recordings showed that after blocking the AMPA receptors by CNQX, the mEPSC frequency dropped from 1.14 ± 0.12 Hz to 0.09 ± 0.02 Hz (n = 18, *p* < 0.01), GDF-15 incubation did not induce a significant increase of frequency (0.14 Hz ± 0. 03 Hz, n = 10, *p* = 0.12). On the other hand, while blocking NMDA receptor by APV did not significantly reduce the mEPSC frequency (1.13 ± 0.11 Hz, n = 18; *p* = 0.96), 30 ng/mL GDF-15 incubation for 1 h increased the frequency of mEPSC by 33.2% (from 1.13 ± 0.11 Hz to 1.52 ± 0.12 Hz, n = 18 and 15, *p* = 0.031). The amplitude and decay time remained unchanged in presence of APV with or without the GDF-15 (Fig. 1F). The data suggested that in the presence of 1.5 mM Mg^2+^ in ACSF and holding potential at −70 mV, mEPSC in the absence or presence of GDF-15 is mainly mediated by AMPA receptors in the pyramidal neurons of mPFC.

**Figure 2:**
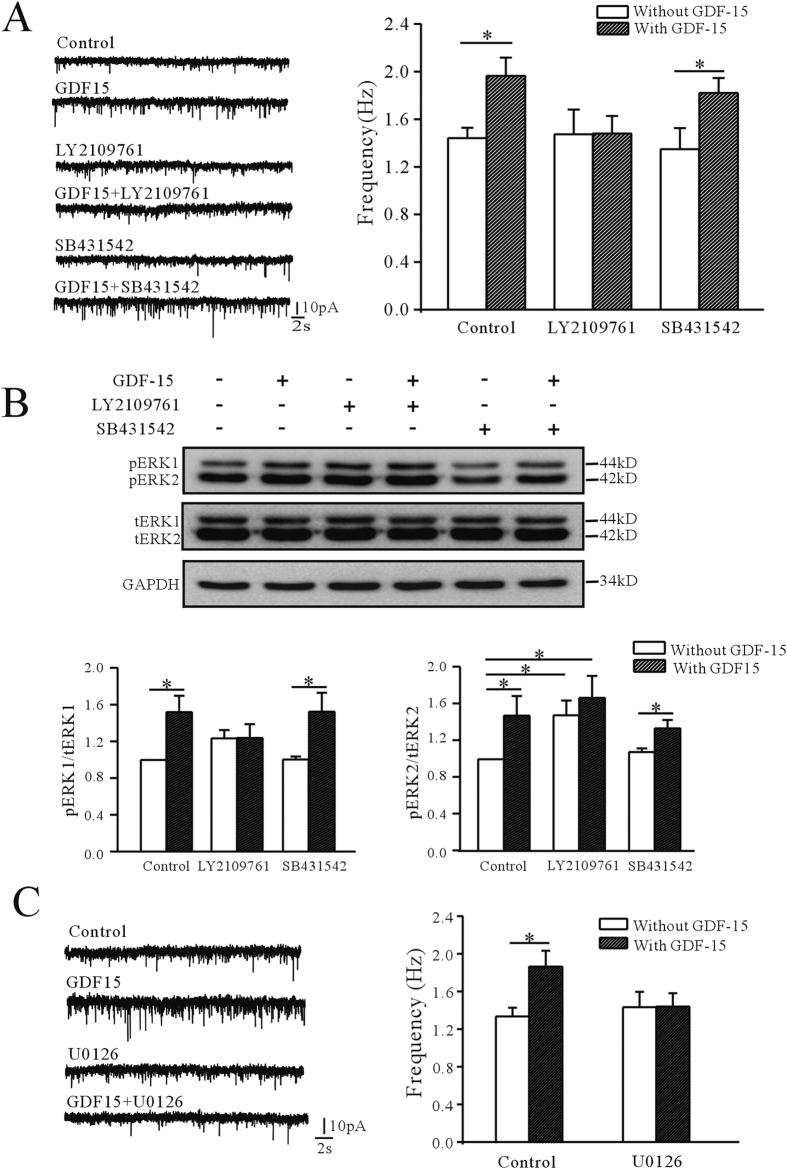
Involvement of TβRII/ERK pathway in GDF-15-induced increase in mEPSC frequency. (**A**) Effects of TβRI/TβRII dual inhibitor LY2109761 and TβRI inhibitor SB431542 on GDF-15-induced up-regulation of mEPSC frequency. (**B**) Representative western blot and bar graph showing effect of GDF-15 on phosphorylated ERK1/ERK2 levels in the absence and presence of LY2109761 and SB431542. pERK1 and pERK2 levels were normalized by total ERK1 and ERK2 levels. (**C**) Representative recordings and bar graph showing the effect of GDF-15 on mEPSC frequencies in the absence and presence of MEK inhibitor U0126. Results are shown as means ± SEM. *p < 0.05 compared with corresponding control (without GDF-15) determined by unpaired Student’s *t-*test.

The TβRI/TβRII heterodimer is a cell surface receptor complex which can be successively activated by the TGF-β subfamily to achieve cross-membrane signaling^14^,^15^. We previously showed that GDF-15 up-regulated expression of the K_V_2.1 α-subunit in rat CGNs through a non-Smad-dependent and TβRII-mediated ERK1/2 pathway^13^. In this study, we therefore used the TβRI/TβRII dual inhibitor LY2109761, and the TβRI (ALK5) specific inhibitor SB431542 to investigate if the TβRII was associated with the effect of GDF-15 on mEPSC frequency in mouse mPFC neurons. Inhibition of TβRI by SB431542 (10 μM) did not eliminate the GDF-15-induced increase in mEPSC frequency (Fig. 2A), which was still significantly increased by 34.8% (from 1.35 ± 0.18 Hz with SB431542 alone, n = 15, to 1.82 ± 0.13 Hz with SB431542 plus GDF-15, n = 16, *p* = 0.040). In contrast, inhibition of TβRI/TβRII with LY2109761 (10 μM) almost eliminated the effect of GDF-15 on mEPSC frequency, which was reduced to 0.48% (n = 19, *p* = 0.980) (Fig. 2A).

We previously showed that exposure of CGNs to GDF-15 markedly induced the phosphorylation of ERK within 60 min^13^. We therefore examined if the ERK signaling pathway was activated by GDF-15 in mPFC by measuring the levels of pERK. Phosphorylation of both ERK1 (44 kDa) and ERK2 (42 kDa) were significantly increased by GDF-15, as shown by western blotting (Fig. 2B). Treatment of mPFC slices with GDF-15 for 60 min resulted in increases of pERK1 and pERK2 levels relative to untreated controls of 151.77 ± 17.8% (n = 6, *p* = 0.016) and 147.35 ± 21.2% (n = 6, *p* = 0.048), respectively (Fig. 2B). Consistent with the results for mEPSC frequency, blocking TβRI alone with SB431542 did not reduce the GDF-15-induced increase in pERK1 and pERK2 levels, which were still increased by 53.82% (n = 5, *p* = 0.039) and 23.76% (n = 6, *p* = 0.027), respectively (Fig. 2B). However, inhibition of TβRI/TβRII with LY2109761 reduced GDF-15-induced phosphorylation of ERK1 and ERK2 to 0.2% (n = 5, *p* = 0.992) and 12.7% (n = 5, *p* = 0.531), respectively (Fig. 2B).

The effects of GDF-15 on ERK activation were confirmed using the specific MEK inhibitor U0126. Incubation with U0126 completely abolished the GDF-15-induced effects on mEPSC frequency (Fig. 2C). In the presence of U0126 (10 μM), GDF-15 only increased mEPSC frequency by 4.06% (n = 15, *p* = 0.799) (Fig. 2C).

These data indicated that GDF-15 activated the TβRII /ERK pathway, which was required for GDF15-mediated enhancement of mEPSC frequency.

### GDF15 enhanced mEPSC frequency in mPFC by increasing glutamate release

**Figure 3:**
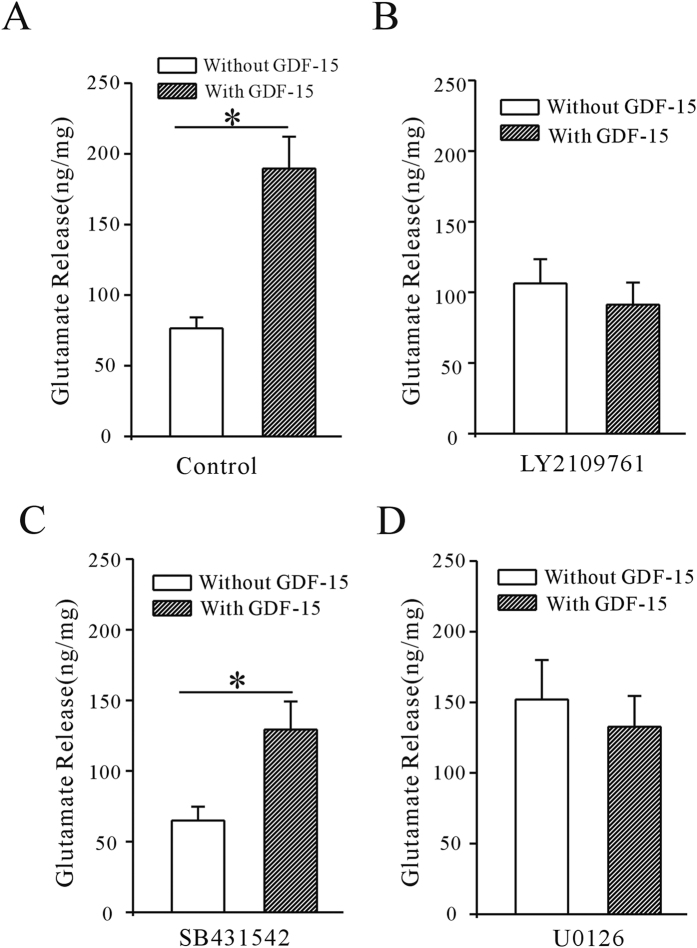
GDF-15 increased glutamate release from mPFC slices incubated in ACSF incubation. (**A**) GDF-15 increased glutamate release, as measured by HPLC. Glutamate concentration was normalized against protein concentration. (**B**–**D**) Effects of LY2109761, SB431542, and U0126 on GDF-15-induced up-regulation of glutamate concentration in ACSF. Results are shown as means ± SEM. *p < 0.05 compared with corresponding control (without GDF-15) determined by unpaired Student’s *t*-test.

It is generally accepted that changes in mEPSC frequency may be caused by modulation of presynaptic transmission, while changes in amplitude or decay time would suggest a different postsynaptic receptor function. We used HPLC to measure the glutamate concentration in the ACSF to confirm the effect of GDF-15 on glutamate release. Slices from the right and left hemispheres were incubated with ACSF in the presence or absence of GDF-15, and glutamate release was measured as ng/mg. GDF-15 significantly increased glutamate release in mPFC slices by 147.05% (from 76.52 ± 7.70 to 189.60 ± 22.57 ng/mg, n = 12, *p* < 0.0001) (Fig. 3A). Consistent with the results for mEPSCs, this increase in glutamate release was reduced by LY2109761 and U0126, to 14.25% (n = 6, *p* = 0.526) and 12.71% (n = 6, *p* = 0.597), respectively (Fig. 3B,D). However, in the presence of SB431642, GDF-15 still increased glutamate release by 99.03% (from 64.95 ± 9.8 to 129.27 ± 19.9 ng/mg, n = 8, *p* = 0.0116) (Fig. 3C).

Overall, these results suggested that GDF-15 enhanced mEPSCs by increasing glutamate release in the mPFC, and activation of the TβRII/ERK pathway is required for the GDF15-mediated increase in glutamate release.

### GDF-15 increased the release of glutamate through T-type calcium channels

**Figure 4:**
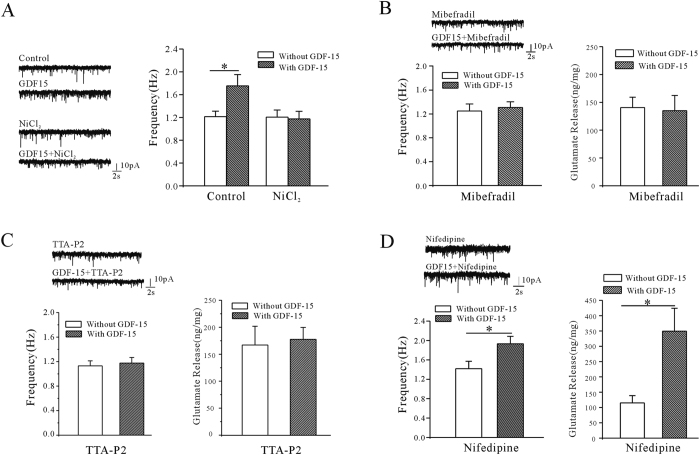
Effects of T-type and L-type VGCC inhibitors on GDF-15-induced enhancement of mEPSC frequency and glutamate release. (**A**) Representative recordings and bar graph showing effect of T-type VGCC blocker NiCl_2_ on GDF-15-induced increase in mEPSC frequency. (**B**) Representative recordings and bar graph showing effect of T-type VGCC blocker mibefradil on GDF-15-induced increases in mEPSC frequency and glutamate release. (**C**) Representative recordings and bar graph showing the effects of T-type VGCC blocker TTA-P2 on the GDF-15-induced increase in mEPSC frequency and glutamate release. (**D**) Representative recordings and bar graph showing effect of L-type VGCC blocker nifedipine on GDF-15-induced increases in mEPSC frequency and glutamate release. Results are shown as means ± SEM. *p < 0.05 compared with corresponding control (without GDF-15) determined by unpaired Student’s *t*-test.

Neurotransmitter release is thought to be triggered by enhanced presynaptic calcium levels associated with voltage-gated calcium channels (VGCCs)^16^,^17^. Growing evidence suggests that T-type VGCCs play a key role in controlling neurotransmission near the rest potential and sustaining neurotransmitter release during mild stimulation^18^. We therefore investigated the association between T-type VGCCs and the GDF-15-induced increase in glutamate release using a specific calcium channel blocker. Treatment of mPFC slices with the T-type VGCC blocker NiCl_2_ (100 μM) significantly eliminated the GDF-15-induced increase in mEPSC frequency to −2.3% (n = 18, *p* = 0.871) (Fig. 4A). Similar results were obtained with different T-type VGCC inhibitors, mibefradil (10 μM) and TTA-P2 (2 μM), which reduced the GDF-15-induced increases in mEPSC frequency to 4.6% (n = 17, *p* = 0.733) and 3.5% (n = 13, *p* = 0.71), respectively (Fig. 4B,C). Mibefradil and TTA-P2 also inhibited the effect of GDF-15 in glutamate release, the GDF-15 induced increase of the glutamate release was lowered from 129.2% to 1.71% (n = 7, *p* = 0.96) or 5.98% (n = 8, *p* = 0.80) when co-incubated the GDF-15 with mibefradil or TTA-P2, respectively (Fig. 4B,C). Because mibefradil is known to also partly inhibit L-type VGCCs^19^, we used nifedipine to exclude a potential role of these L-type VGCCs. The GDF-15-enhanced mEPSC frequency and glutamate release were unaffected by nifedipine (10 μM) (Fig. 4D), and GDF-15 increased the mEPSC frequency by 36.2% (from 1.42 ± 0.15 Hz to 1.93 ± 0.15 Hz, n = 16 and 21, *p* = 0.025) and enhanced glutamate release by 203.5% (from 115.13 ± 23.72 to 349.42 ± 74.87 ng/mg, n = 6, *p* = 0.013) in the presence of nifedipine (Fig. 4D), suggesting that L-type VGCCs do not play a major role in the GDF-15-mediated effects on mEPSC frequency and glutamate release.

**Figure 5:**
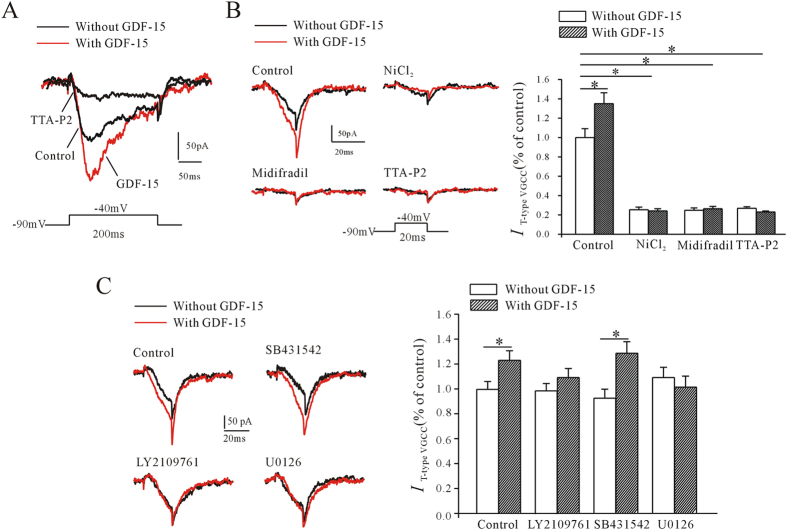
Effect of GDF-15 on T-type VGCC current (*I*_T-type VGCC_) in pyramidal neurons in mPFC. (**A**) *I*_T-type VGCC_ was elicited in the presence of TTX (1 μM) and nifedipine (10 μM), neuron was held at −90 mV and depolarized to −40 mV for 200 ms. Representative recordings show the effects of TTA-P2 and GDF-15 on *I*_T-type VGCC_ amplitude. (**B**) To avoid the overlap of R-type calcium channels, *I*_T-type VGCC_ was recorded by depolarizing to −40 mV for 20 ms, and the tail currents following the end of the depolarizing pulse were measured. NiCl_2_, mibefradil and TTA-P2 inhibited *I*_T-type VGCC_. Left panel shows representative recordings, right panel shows bar graph of the effects of NiCl_2_, mibefradil and TTA-P2 on *I*_T-type VGCC_ amplitude. (**C**) Representative recordings and bar graph showing effects of LY2109761, SB431542, and U0126 on GDF-15-induced increase of *I*_T-type VGCC._ Results are shown as means ± SEM. *p < 0.05 compared with corresponding control (without GDF-15) determined by unpaired Student’s *t*-test.

To confirm the role of T-type VGCCs in GDF-15-induced enhancement of mEPSC frequency and glutamate release, we observed the effect of GDF-15 on T-type VGCC currents (*I*_T-type VGCC_) in pyramidal neurons in mPFC slices directly. *I*_T-type VGCC_ was recorded in the presence of TTX (1 μM) and nifedipine (10 μM), neuron was held at −90 mV and depolarized to −40 mV for 200 ms. The currents recorded could be mostly inhibited by 2 μM TTA-P2 (Fig. 5A) and were increased after the incubation of GDF-15 for 60 min (Fig. 5A). To avoid possible overlap with R-type calcium channels, the tail currents following the end of the brief depolarizing pulse (20 ms) were measured^20^,^21^. NiCl_2_ (100 μM), mibefradil (10 μM) and TTA-P2 (2 μM) significantly inhibited the tail currents by 74.6 ± 1.0% (n = 13, *p* < 0.001), 75.2 ± 2.4% (n = 12, *p* < 0.001) and 72.9 ± 1.6% (n = 10, *p* < 0.001), respectively (Fig. 5B), indicating that the recorded currents were mostly *I*_T-type VGCC_. The same result was found that GDF-15 incubation for 60 min significantly increased the T-type calcium channel mediated tail current by 34.8 ± 11.2% (n = 15, *p* = 0.024) (Fig. 5B). And in the presence of the NiCl_2_ (100 μM), mibefradil (10 μM) or TTA-P2 (2 μM) respectively, GDF-15 no longer increase the *I*_T-type VGCC_ (Fig. 5B). Consistent with the results for mEPSCs and pERK, the GDF-15-induced increase in *I*_T-type VGCC_ was eliminated by the TβRI/TβRII dual inhibitor LY2109761, but not by the TβR1 inhibitor SB431542 (Fig. 5C). In the presence of LY2109761 (10 μM) and SB431542 (10 μM), GDF-15 increased *I*_T-type VGCC_ by 10.32% (n = 18, *p* = 0.272) and 38.76% (n = 23, *p* = 0.007), respectively. Similarly, U0126 (10 μM) inhibited the effect of GDF-15 on *I*_T-type VGCC_, and reduced the increase to -7.75% comparing with U0126 alone (n = 15, *p* = 0.517) (Fig. 5C).

Overall, these data indicated that GDF-15 increased T-type VGCC activity via the TβRII/ERK pathway.

### GDF-15 increased T-type VGCC activity through promoting Ca_V_3.1 and Ca_V_3.3 surface expression

**Figure 6:**
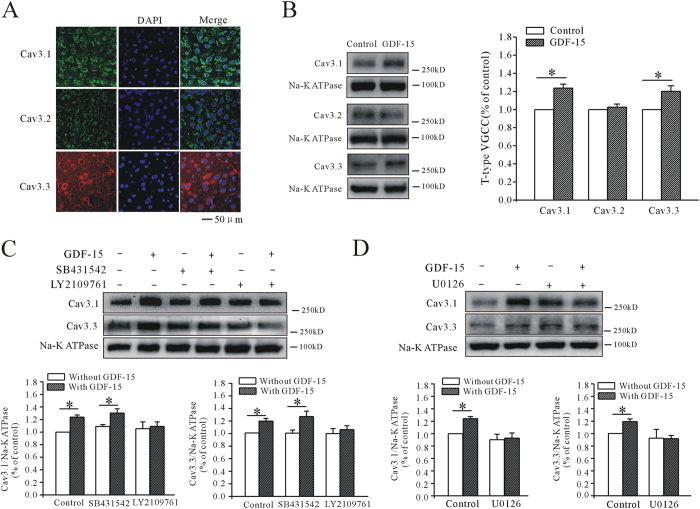
GDF-15 enhanced Ca_V_3.1 and Ca_V_3.3 surface expression on pyramidal neurons in mPFC. (**A**) Microscopic confocal images showing expression of Ca_V_3.1, Ca_V_3.2 and Ca_V_3.3 α-subunits of T-type VGCCs on pyramidal neurons in mPFC. Bar = 50 μm. (**B**) Western blot showing effects of GDF-15 on T-type VGCC surface expression. Na^+^/K^+^-ATPase was used as loading control. (**C**,**D**) Western blot showing effects of LY2109761, SB431542, and U0126 on GDF-15-induced enhancement of Ca_V_3.1 and Ca_V_3.3 surface expression. Results are shown as means ± SEM. *p < 0.05 compared with control (without GDF-15) determined by unpaired Student’s *t*-test.

The effect of GDF-15 on mEPSCs was short-term, and 1 h was not long enough to influence protein transcription and translation. We therefore suspected that GDF-15 may promote the membrane trafficking of T-type VGCC proteins. Using specific antibodies, we confirmed that all three α-subunits of T-type VGCCs (Ca_V_3.1, Ca_V_3.2, and Ca_V_3.3) were expressed on mPFC pyramidal neurons (Fig. 6A), but Ca_V_3.1 was the most highly expressed and Ca_V_3.2 the least expressed, consistent with a previous *in situ* hybridization study^22^. We then measured the effect of GDF-15 on the surface expression of T-type VGCCs using a membrane extraction kit. Western blotting showed that GDF-15 significantly increased the membrane expression of Ca_V_3.1 and Ca_V_3.3 by 23.63 ± 4.49% (n = 9, *p* < 0.0001) and 19.94 ± 6.18% (n = 9, *p* = 0.005), respectively, but had no significant effect on Ca_V_3.2 (Fig. 6B). These results suggest that Ca_V_3.1 and Ca_V_3.3 membrane expression levels were up-regulated by GDF-15.

Further, we investigated the role of the TβRII/ERK pathway in the GDF-15-induced up-regulation of Ca_V_3.1 and Ca_V_3.3 using the corresponding inhibitors. Co-incubation of PFC slices with GDF-15 and LY2109761 (10 μM) reduced the up-regulation of Ca_V_3.1 and Ca_V_3.3 membrane expression to 3.43% (n = 7, *p* = 0.785) and 6.16% (n = 7, *p* = 0.555), respectively, which were significantly different from the results with GDF-15 alone (Fig. 6C). As expected, SB431542 had no influence on the effects of GDF-15 on membrane expression. GDF-15 still up-regulated Ca_V_3.1 and Ca_V_3.3 membrane expression by 19.45% (n = 5, *p* = 0.023) and 26.76% (n = 7, *p* = 0.031), respectively, in the presence of SB431542 (10 μM). Similarly, U0126 (10 μM) significantly inhibited the GDF-15-induced up-regulation of Ca_V_3.1 and Ca_V_3.3 expression to 3.56% (n = 5, *p* = 0.801) and -0.99% (n = 5, *p* = 0.95), respectively (Fig. 6D).

**Figure 7:**
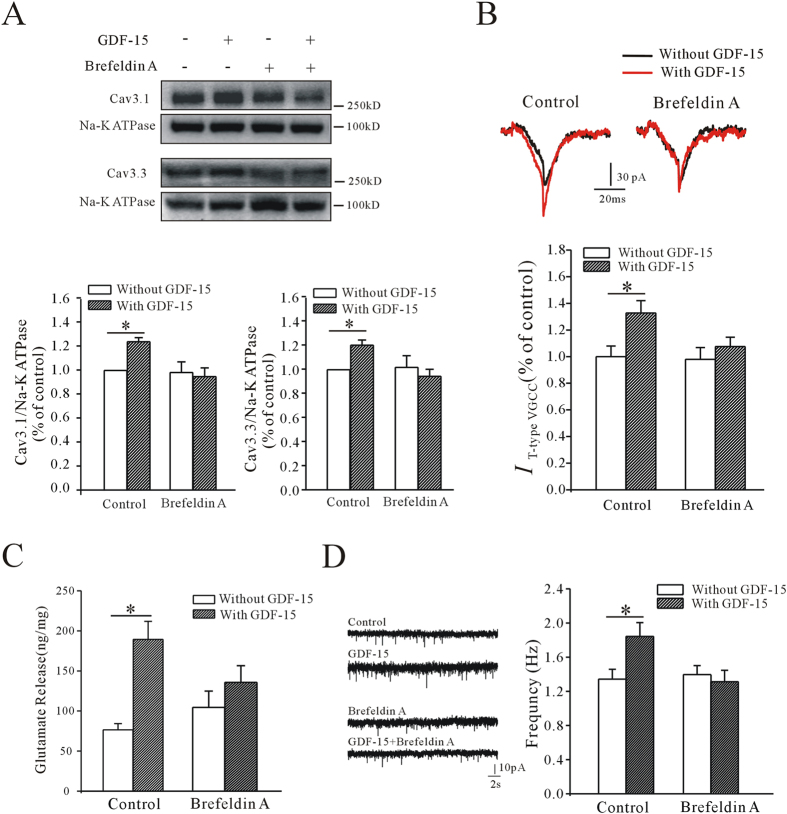
Effects of brefeldin A on GDF-15-induced mEPSC frequency, glutamate release, Ca_V_3.1 and Ca_V_3.3 surface expression, and *I*_T-type VGCC_ amplitude. (**A**) Western blot showing effects of brefeldin A on GDF-15-induced Ca_V_3.1 and Ca_V_3.3 surface expression. (**B**) Effect of brefeldin A on GDF-15-induced increase in *I*_T-type VGCC_. (**C**) Bar graph showing effect of brefeldin A on GDF-15-induced glutamate release. (**D**) Representative recordings and bar graph showing that brefeldin A eliminated the effect of GDF-15 on mEPSC. Results are shown as means ± SEM. *p < 0.05 compared with control (without GDF-15) determined by unpaired Student’s *t*-test.

Brefeldin A is a lactone antibiotic produced by fungi, which can indirectly inhibit protein transport from the endoplasmic reticulum to the Golgi apparatus by preventing formation of coat protein I-mediated transport vesicles^23^,^24^. We used brefeldin A to determine if the effects of GDF-15 on *I*_T-type VGCC_ and the surface expression of Ca_V_3.1 and Ca_V_3.3, as well as the subsequent increases in glutamate release and mEPSC frequency, were caused by Ca_V_3.1 and Ca_V_3.3 protein trafficking. Incubation with brefeldin A alone for 1 h had no effect on the surface expression of Ca_V_3.1 and Ca_V_3.3, suggesting no effect on T-type VGCCs already present in the membrane (Fig. 7A). However, co-exposure of brain slices to GDF-15 and brefeldin A (10 μM) inhibited the increase in membrane expression of T-type VGCC protein; membrane expression levels of Ca_V_3.1 were increased by −3.65% (n = 8, *p* = 0.76) and levels of Ca_V_3.3 by −7.99%, compared with controls (n = 6, *p* = 0.521) (Fig. 7A). Similarly, GDF-15 failed to increase *I*_T-type VGCC_ in the presence of brefeldin A, and the *I*_T-type VGCC_ was increase by 9.87% compared with brefeldin A alone (n = 26, *p* = 0.394) (Fig. 7B). HPLC analysis indicated that brefeldin A inhibited the GDF-15-induced increase in glutamate release (from 147.05% with GDF-15 alone to 29.5% with GDF-15 plus brefeldin A, n = 6, *p* = 0.314) (Fig. 7C). The frequency of mEPSCs in the presence of GDF-15 and brefeldin A was 1.31 ± 0.14 Hz (n = 22), which was similar to that in the presence of brefeldin A alone (1.39 ± 0.11, n = 26, *p* = 0.623) (Fig. 7D).

Overall, these results indicated that GDF-15 increased glutamate release through promoting T-type VGCC trafficking to the membrane.

## Discussion

GDF-15 is known to play pivotal roles in neuroprotection, neural regeneration, and axonal elongation^7^,^12^,^25^; however, little is known about its precise function in neuronal excitability, its mechanism of action, and its downstream effectors. We previously showed that incubation of CGNs with GDF-15 activated TβRII and phosphoinositide 3-kinase/Akt/mammalian target of rapamycin (mTOR) signaling to increase *I*_K_ amplitude and K_V_2.1 expression, with possible developmental significance^13^. In the current study, incubation of cortical neurons with GDF-15 for 60 min up-regulated expression levels of the Ca_V_3.1 and Ca_V_3.3 subunits of T-type VGCC on the membrane, thereby increasing glutamate release and the frequency of mEPSCs, involving activation of the same receptor and downstream signaling components as those previously reported in CGNs^13^.

TGF-β superfamily ligands mediate their effects via the transmembrane TβRI and TβRII receptor heterodimer^14^,^26^. There is good evidence to indicate that activation of TGF-β receptors not only activates the Smad signaling pathway, but also non-Smad pathways, such as p38, JNK/MAPK, mTOR and Ras-ERK^26^,^27^,^28^. We previously found that the Akt/mTOR and MAPK/ERK pathways were activated in CGNs by GDF-15 treatment, though activation of ERK signaling was not required for GDF-15-induced transcriptional regulation of K_V_2.1 expression^13^. Use of pharmacological inhibitors demonstrated that activation of TβRII was associated with the effect of GDF-15 on cortical neurons; similar to the results in CGNs, but activation of ERK signaling was also required for GDF-15-induced up-regulation of Ca_V_3.1 and Ca_V_3.3 expression. This apparent difference is likely because GDF-15 up-regulated K_V_2.1 expression in CGNs at the Akt/mTOR-mediated transcriptional level, but induced increases in Ca_V_3.1 and Ca_V_3.3 expression at MAPK/ERK-associated posttranslational levels, similar to the Hu *et al*. report, in which MAPK/ERK regulated the *I*_A_ channel by direct phosphorylation of K_V_4.2 subunits^29^. We also noted that there is a significant increase in pERK2 level in LY2109761 treated slice, suggesting that blocking TβRI/TβRII with LY2109761 for 1 h is capable of activating ERK2 phosphorylation via an unknown pathway in mPFC. It is likely because TGF-β receptors mediate both of Smad and non-Smad signal pathways, the crosstalk with other downstream signaling may activate ERK2. However, this effect did not affect LY2109761’s role on inhibiting GDF-15-induced the increase of pERK1/2 through blocking TβRI/TβRII.

VGCCs are voltage sensors that convert membrane depolarization into intracellular Ca^2+^ signals. In neurons, VGCCs include L-, N-, P/Q-, R-, and T-type Ca^2+^ channels^30^,^31^. T-type VGCCs are transient, low-voltage activated Ca^2+^ channels that control Ca^2+^ entry during depolarization near resting potential^32^. Increasing evidence suggests that T-type VGCCs are loosely coupled to neurotransmission near the resting potential and sustain neurotransmitter release during mild stimulation^18^. In our study, the T-type VGCC blockers NiCl_2_, mibefradil and TTA-P2 eliminated the increases in mEPSC frequency and glutamate release induced by GDF-15, suggesting the involvement of T-type VGCCs. However, NiCl_2_, mibefradil and TTA-P2 alone did not affect the frequency of mEPSCs in cortical neurons under control conditions. It is possible that a large percentage of the T-type calcium channels are tonically inactivated at normal neural resting membrane potentials^33^,^34^,^35^,^36^,^37^, and only a small proportion of channels remains tonically activated at membrane potentials within the window current^38^. We therefore hypothesized that mibefradil and NiCl_2_ only inhibited the fraction of T-type VGCCs that were increased by GDF-15. This phenomenon is consistent with the situation in which the contribution of T-type channels is indirect, and requires either the activation of coupled presynaptic receptors or depolarization of the membrane potential by blocking the ion channels^39^,^40^. A previous study found that mEPSCs in striatopallidal medium spiny neurons were mediated by the Ca_V_1.3α1 subunit of L-type VGCCs^41^. This difference with the present study may be attributable to different neuron types, different developmental states, and/or the different animals used. In addition, we have noticed that recent study from the juvenile mice calyx of Held synapse and neocortical neurons indicated that spontaneous glutamate release can be triggered indirectly by the Ca^2+^ entry through VGCCs and be mediated via a different Ca^2+^ -sensing mechanism^42^,^43^, whether GDF-15-induced the increase of glutamate release is associated with those Ca^2+^ -sensing mechanism is worthy of further study.

Three genes, *CACNA1G*, *CACNA1H*, and *CACNA1I*, have been identified as coding for the T-type VGCC subunits Ca_V_3.1/α1G, Ca_V_3.2/α1H, and Ca_V_3.3/α1I, respectively^34^. The biophysical properties, structure–function relationships, and divergent physiological roles of the three Ca_V_3 channels of T-type VGCCs have been documented^33^,^44^,^45^. Moreover, previous research has focused more on the structure and function of Ca_V_3.2 rather than Ca_V_3.1, Ca_V_3.3^46^,^47^. However, although all three Ca_V_3 subunits were detected in mPFC neurons by immunofluorescence, Ca_V_3.2 was the least expressed, consistent with a previous *in situ* hybridization study^22^. More than this, GDF-15 mainly increased the expression of Ca_V_3.1 and Ca_V_3.3, rather than Ca_V_3.2. T-type VGCCs are known to lack an α-interaction domain and are thus unable to interact with the β-subunit, which usually controls trafficking of the other VGCCs to the plasma membrane^48^. The intracellular loop connecting repeats I and II (I–II loop) of T-type VGCCs is thus an important regulator for trafficking, with distinct effects on the three channel types^44^,^49^,^50^. In addition to the lower expression of Ca_V_3.2 compared with Ca_V_3.1 and Ca_V_3.3 in the mPFC, the selective up-regulation of Ca_V_3.1 and Ca_V_3.3 by GDF-15 may be associated with differences in structural properties and trafficking mechanisms among the different Ca_V_3 subunits. Further studies are needed to clarify the precise mechanisms.

As membrane proteins, the expression of functional ion channel subunits can be modulated at multiple levels, including transcription, translation and trafficking. Long-term up-regulation of protein expression is mainly associated with transcription and translation^21^,^51^, while short-term modulation of ion-channel densities may be the result of rapid mechanisms involving changes in intracellular trafficking of channel proteins^52^. In this study, treatment with GDF-15 for 1 h was enough to enhance the surface expression of Ca_V_3.1 and Ca_V_3.3, suggesting the involvement of a short-term modulatory mechanism. This speculation that GDF-15 up-regulate Ca_V_3.1 or Ca_V_3.3 surface expression by activation of ERK-mediated trafficking was supported by the effect of the protein-transport inhibitor, brefeldin A. Although ERK-mediated protein trafficking is known to play a role in the regulation of T-type VGCC expression^24^,^53^,^54^, the opposite regulatory effect has also been found in L-type VGCC protein^55^. In addition, a recent study identified the actin-binding protein Kelch-like 1 as a regulator of T-type VGCC protein, responsible for enhanced cell surface expression^56^. However, the mechanisms whereby ERK up-regulates Ca_V_3.1 or Ca_V_3.3 trafficking, and the role of Kelch-like 1 in the GDF-15-mediated effect on increased cell surface expression of Ca_V_3.1 or Ca_V_3.3 remain to be elucidated.

Both electrophysiological and behavioral studies have suggested that the mPFC may be involved in recognition memory^57^. mPFC neurons have been shown to carry information concerning the relative familiarity of individual stimuli^58^,^59^. In our study, short-term application of GDF-15 significantly enhanced neurotransmitter release and mEPSCs in mPFC neurons, indicating a previously unreported role for GDF-15 in rapidly regulating neuronal excitability. It has been noted that GDF-15 mRNA and protein levels were dramatically up-regulated at the sites of cryolesions or ischemic lesions^6^,^12^, suggesting that GDF-15 may not only promote survival and protect neurons against lesions, but may also affect neuronal excitability or synaptic activity in the lesioned region, thus affecting neural-network excitability, and ultimately processes such as learning and memory. Fuchs *et al*. found that GDF-15 levels were associated with cognitive performance and age-related cognitive decline in humans, indicating a negative role for GDF-15 in recognition memory^60^. Further animal behavioral tests after overexpression of GDF-15 in different brain areas are needed to determine the physiological and pathophysiological effects of GDF-15 on recognition memory.

In conclusion, the results of this study demonstrated that GDF-15 increased release of the neurotransmitter glutamate in mPFC pyramidal neurons via posttranscriptional regulation of Ca_V_3.1 and Ca_V_3.3 trafficking. Furthermore, the same signaling pathways and receptors identified in CGNs were activated by GDF-15 in pyramidal neurons. T-type currents occur in neurons throughout the brain, with particularly large currents in the thalamic, septal, and sensory neurons. Their preferential localization in dendrites suggests that T-type channels play an important role in synaptic integration^34^. This study thus provides an important insight into the mechanisms underlying the functions of GDF-15 in the brain.

## Materials and Methods

### Experimental animals

Female C57BL/6 mice 3–4 weeks old (15–20 g) were purchased from Slac Laboratory Animals (Shanghai, China) and housed under a 12-h light/dark cycle, with food and water available ad libitum. All the experiments were performed in accordance with the National Institutes of Health (NIH) Guidelines for the Care and Use of Laboratory Animals. The protocol was approved by the Committee on the Ethics of Animal Experiments of Fudan University (permit number: 20090614-001). Efforts were made to minimize the number of animals used and their suffering.

### Slice preparation and whole-cell recording

Mice were anesthetized with sodium pentobarbital and then decapitated. The brain was quickly removed into cold, pre-oxygenated cutting solution containing 220 mM sucrose, 3 mM KCl, 5 mM MgCl_2_, 1 mM CaCl_2_, 1.25 mM NaH_2_PO_4_, 26 mM NaHCO_3_, and 10 mM glucose. The brain was glued to the stage and submerged in the cold cutting solution, and 200-μm slices containing the mPFC were cut coronally from the PFC using a vibratome slicer (DOSAKA, Kyoto, Japan). About three slices per hemisphere were placed into ACSF at 34 °C to recover for at least 1 h, and bubbled with 95% O_2_–5% CO_2_, before recording. ASCF contained 125 mM NaCl, 2.5 mM KCl, 1.5 mM MgSO_4_, 2.5 mM CaCl_2_, 1 mM NaH_2_PO_4_, 26 mM NaHCO_3_, and 10 mM glucose.

Whole-cell recordings were made according to standard procedures, at room temperature^61^. PFC slices were transferred to a recording chamber and continuously perfused with oxygenated ACSF. mEPSCs were recorded from pyramidal cells in layers II/III using an Axon 700B amplifier (Molecular Devices, Union City, California, USA) under visual control, using differential interference contrast and infrared optics via a water-immersion objective (Olympus, Tokyo, Japan) and a CCD camera (Qimaing, Surrey, Canada). The recording pipettes were 3–5 MΩ filled with intracellular solution containing 150 mM K^+^ -gluconate, 0.4 mM EGTA, 8 mM NaCl, 2 mM ATP-Mg, 0.1 mM GTP-Na, and 10 mM HEPES. Signals were low-pass filtered at 2 kHz and digitized at 5 kHz using Digidata 1322 and 1440 (Molecular Devices, Sunnyvale, California, USA). mEPSCs were recorded at a holding potential of −70 mV in the presence of tetrodotoxin (TTX) (1 μM) and bicuculline (10 μM) to block voltage-dependent sodium-channel-mediated spontaneous action-potential generation and GABA-mediated inhibitory postsynaptic current receptors, respectively. For T-type calcium channel recording, the extracellular solution contained 20 mM tetraethylammonium (TEA), 115 mM sodium methanesulfonate, 3.5 mM KCl, 10 mM HEPES, 2 mM CaCl_2_, 2 mM MgCl_2_, 4 mM 4 - aminopyridine (4-AP), and 25 mM glucose. The intracellular solution contained 125 mM CsCl, 1 mM MgCl_2_, 1 mM CaCl_2_, 10 mM HEPES, 10 mM EGTA, 3 mM Mg-ATP, and 0.3 mM Tris-GTP. Series resistance (Rs) was monitored during recording. Cells in which the Rs varied by >20% and the Rs was >60 MΩ were excluded from subsequent analyses. mEPSCs and *I*_T-type VGCC_ were collected using pClamp10.2 software (Molecular Devices) and analyzed using the Mini-analysis program (Synpatosoft, Decatur, Georgia, USA) and Clampfit 9.2 (Molecular Devices), respectively.

### High-performance liquid chromatography

Brain slices were incubated with GDF-15 or inhibitors in ACSF bubbled with 95% O_2_–5% CO_2_ for 1 h. The ACSF was then collected and frozen at −80 °C for later analyses. The slices were homogenized in ice lysis buffer containing protease inhibitor (Sigma, St Louis, Missouri, USA), rotated on ice for 40 min, and centrifuged at 13,800 × *g* for 20 min at 4 °C. The supernatants were collected to measure the protein concentrations. The samples were subjected to reversed-phase HPLC (ThermoFisher, Waltham, Massachusetts, USA) with fluorometric detection following pre-column derivatization with o-phthalaldehyde to analyze glutamate concentrations, as described previously^62^. Chromatography was performed on a reversed-phase C-18 column using a pH sodium acetate methanol gradient. Methionine sulfone was added to each sample as an internal standard. External standards containing 40, 400, or 4000 pmol/20 ml glutamate were run at the beginning and end of every group. The peak heights of glutamate were initially normalized to the methionine sulfone peak and then quantified according to the linear relationship between peak height and the amounts of the corresponding standards. Glutamate release was normalized by the total protein in each single brain slice, and expressed as ng/mg protein^63^.

### Immunohistochemistry

Mice were anesthetized with sodium pentobarbital and perfused transcardially with normal saline followed by 4% paraformaldehyde. Brains were post-fixed in the 4% paraformaldehyde overnight at 4 °C. Coronal brain sections (40 μm) were cut using a vibratome slicer (Leica, Wetzlar, Germany) and processed for immunofluorescence.

Sections were transferred into 0.5 ml of blocking solution (5% bovine serum albumin; 0.5% Triton X-100, and 0.05% sodium azide in PBS) in a multi-well plate, placed on a shaker and shaken gently at room temperature for 1.5–2 h. The blocking solution was then replaced with the following antibody solutions for 2 days at 4 °C: mouse anti-Ca_V_3.1 (1:50, NeuroMab, Davis, California, USA), mouse anti-Ca_V_3.2 (1:50, NeuroMab), and rabbit anti-Ca_V_3.3 (1:100, Santa Cruz, Dallas, Texas, USA) in 1% bovine serum albumin, 0.5% Triton X-100, and 0.05% sodium azide in PBS. After 2 days, the antibody solution was removed and the sections were washed with PBST (0.1% Triton X-100 in PBS) three times for 10 min, with a final wash for 4–5 h. The sections were incubated with secondary antibody solution (FITC-labeled goat anti-mouse IgG, Cy3-labeled goat anti-rabbit IgG, 1:500) overnight at 4 °C. The antibody solution was replaced with PBST and the sections were washed three times for 10 min each in PBS. DAPI was added to the slices to stain the nucleus. All sections were covered with coverslips using an anti-fade mounting medium, and then observed under a Leica SP2 confocal laser scanning microscope. Immunoreactivity was examined at optimal resolution. Confocal photomicrographs were further processed to adjust scaling, brightness, and contrast.

### Western blotting

Mice were anesthetized with sodium pentobarbital and decapitated. The brain was removed, sectioned, and incubated as described previously for total protein extraction. After incubation, the slices were homogenized in ice lysis buffer containing protease inhibitor (Sigma), rotated on ice for 40 min, and centrifuged at 13,800 × *g* for 20 min at 4 °C. The supernatants were frozen at −80 °C for later western blotting. For membrane-protein extraction, the slices were lysed using Membrane and Cytosol Protein Extraction Kit (Beyotime, Shanghai, China). Before analysis, the supernatant was diluted in sample buffer containing β-mercaptoethanol. Equal amounts of protein were loaded and separated by electrophoresis in 8% SDS-PAGE gels, and transferred onto PVDF membranes (Merck Millipore/Merck KGaA, Darmstadt, Germany). The membranes with proteins were blocked by 10% defatted milk at room temperature for 1 h and then incubated overnight at 4 °C with rabbit antibodies to phosphorylated ERK (pERK) (1:1000, Cell Signaling Technology, Danvers, Massachusetts, USA), rabbit anti-ERK (1:1000, Cell Signaling Technology), mouse anti-Ca_V_3.1 (1:200, NeuroMab), mouse anti-Ca_V_3.2 (1:200, NeuroMab), or rabbit anti-Ca_V_3.3 (1:500, Santa Cruz) primary antibody. After three washes for 10 min each, the protein blots were incubated with secondary goat anti-rabbit IgG conjugated with horseradish peroxidase (1:1000, Kang Cheng, Shanghai, China) for 2 h at room temperature. Signals were finally visualized using enhanced chemiluminescence (ThermoFisher), and the blots were exposed in a gel-imaging analyzer (Bio-Rad, Hercules, California, USA). pERK1/pERK2 was normalized against total ERK1/ERK2 and expressed as fold-increase compared with control. Na^+^/K^+^-ATPase (1:1000, Cell Signaling Technology) was used as an internal control for membrane proteins, to ensure that the total protein levels were equal.

### Data analysis

Data are expressed as mean ± SEM. Differences among multiple groups were analyzed using one-way ANOVA, and differences between two groups using Student’s *t*-tests. A *p* value <0.05 indicated statistical significance.

## Additional Information

**How to cite this article**: Liu, D.-D. *et al.* Growth differentiation factor-15 promotes glutamate release in medial prefrontal cortex of mice through upregulation of T-type calcium channels. *Sci. Rep.* **6**, 28653; doi: 10.1038/srep28653 (2016).
